# Efficacy and experience of system constellations in virtual reality (VR): study protocol for a randomized controlled feasibility study

**DOI:** 10.1186/s40814-024-01513-4

**Published:** 2024-12-12

**Authors:** Tobias van Bebber, Eik-Henning Tappe, Thomas Druyen, Heiko Kleve, Tom Rüsen, Christina Hunger-Schoppe

**Affiliations:** 1 Clinical Psychology and Psychotherapy III, Faculty of Health (Department of Psychology and Psychotherapy), Witten/Herdecke University, Alfred-Herrhausen-Str. 50, Witten, 58455 Germany; 2 Department of Social Work (Teaching and Research Area: Digitization and Media Pedagogy in Social Work), University of Applied Science Münster, Friesenring 32, Münster, 48147 Germany; 3 Witten Institute for Family Business (WIFU), Witten/Herdecke University, Alfred-Herrhausen-Str. 50, Witten, 58455 Germany

**Keywords:** Virtual reality (VR), System constellation, Randomized controlled trial (RCT), Organization, Family, Counseling, Systemic therapy, Feasibility, Pilot, Psychological functioning, Social system functioning

## Abstract

**Background:**

Though evidence-based research about system constellations (SCs) appears marginal, it is applied in many psychiatric, psychological, and psychotherapeutic institutions as well as in different contexts of organizational counseling. To date, only one randomized controlled trial (RCT) has been conducted to assess the short- to long-term efficacy of SCs, which entail clients meeting at the same location in person. This study is to investigate the feasibility of a RCT investigating SCs in virtual reality (VR), and to calculate the number of clients needed for a confirmatory RCT.

**Design:**

We will perform a prospective, monocentric, parallel-group, feasibility RCT with subsequent intervention. A total of 128 clients of 3-day group-based SC-VR seminars will be randomized to either the intervention group (IG; *n* = 64) or wait-list group (WLG; *n* = 64), which receives SCs in VR after 4 months. *Feasibility and acceptability* of the (1) research methodology and (2) intervention as well as the (3) estimation of effect sizes will be assessed using qualitative and quantitative data. Based on the model of a general mental health, the *proposed primary outcome* includes the SC-VR adherence, and the *proposed secondary outcomes* refer to psychological functioning (OQ-45.2), social system functioning (EXIS), psychological distress (FEP-2), motivational incongruence (INK-SF), and goal attainment (GAS). We plan to investigate the short-term efficacy at 2-week and 4-month follow-up within the RCT design (*n* = 128), and mid-term efficacy at 8- and 12-month follow-up for the intervention group (*n* = 64).

**Discussion:**

The study is expected to be the first study on the feasibility of SC-VR. We will reflect on successfully implemented study procedures, and we will provide recommendations for changes considering the design, rationale, analyses, and interpretation of the study results where they became necessary. The discussion will conclude with an evaluation whether a confirmatory RCT on SC-VR is worth the investment of future resources, including the calculated number of clients needed based on the efficacy trends derived from this feasibility study.

**Trial registration:**

ClinicalTrials.gov: ID = N CT05557890; date of registration: September 23, 2022; https://clinicaltrials.gov/ct2/show/NCT05557890.

## Background

Over the past 30 years, many forms of group-based psychosocial interventions, rooted in psychotherapy but defined as group counseling, have emerged [1]. One popular but controversial method is the system constellation (SC). SCs refer to an approach which integrates ideas from organizational and family systems counseling with elements from psychodrama. By externalizing significant elements, SCs depict the inner image someone has of a personally important social system in a visible and tangible way. The aim is to get a more explicit picture of the psychosocial conflict structure and thus be better able to facilitate change. Though evidence-based research about SCs appears marginal, the method itself is applied in many psychiatric, psychological, and psychotherapeutic institutions as well as in different contexts of organizational counseling. The first randomized controlled trial (RCT), the “Heidelberg study on systemic constellations” [2], indicates a short- to long-term efficacy of SCs [2–5]. What most formats of SCs have in common is that they require a group setting including 10 people or more which takes place in presence. The intervention usually takes place within 1- to 3-day seminars [2]. This requires a great logistical, time, and economic investment, which makes it difficult to use SCs regularly in everyday counseling. It also represents a barrier for those who strive for a stronger anonymous setting. A new, expanded and possibly lower-threshold approach for dealing with psychosocial problems might be the virtual reality (VR). This technology is already being used successfully in diverse psychotherapy as well as counseling settings [6]. It however never has been used in the practice of SCs. The purpose of this study is to investigate the feasibility of SCs as an intervention in VR.

### System constellation

There are at least three types of SCs: family and organizational constellations assume systemic laws and a natural order of elements, and aim to release entanglements and increase functioning. Exploratory constellations instead focus on understanding systems and recognizing patterns, and do not aim at solutions or the restoration of elements in an order assumed as natural [7, 8]. SCs, as they will be used in our study, represent spatial arrangements of a social system, e.g., family or organization, in which individuals, who are not members of the real system, serve as stand-ins for the clients’ relationship members [9]. Conceptually, the SC approach is influenced by group and family therapy [10], especially the concept of transgenerational “invisible loyalties” between family members [11]. Technically, it integrates elements from psychodrama [12] by using strangers as stand-ins and from family sculptures [13] by setting up a spatial arrangement to symbolize a social system. One central aim is to identify patterns of systemic dynamics and to change problem-oriented perspectives into a more resource-oriented as well as problem-appreciative understanding of the individual members’ ways of communicating and interacting within the affected social system. Consequently, the social system and its members experience a more related and autonomous relationship.

The procedure for each individual SC is as follows [2–5]: First, the facilitator briefly interviews the client, who takes part in the SC as an active client (AC), about the issue at stake. A decision is made about which system will be set up spatially, e.g., the current organizational team the AC is part of. Next, the AC selects group members to act as stand-ins for the team members including him- or herself, as well as organizational entities (e.g., goals, projects, aspects of the organizational culture). Then, the AC places all stand-ins in a concretely defined space which represents the so-called problem picture. Spatial distances, angles, and body postures are meant to correspond to the ACs’ inner image of the team system in question. Once this initial constellation is set up, the AC takes a seat to observe the process. Initially, the stand-ins do not move, interact, or speak. When asked by the facilitator, they voice perceptions and observations based on their position in the constellation (e.g., “I don’t feel good,” or “This is a good place”) and remark on inter- as well as intrapersonal aspects relating to the others (e.g., “The new project stands between me and the rest of the team”). Based on these statements, and considering theoretical principles of SCs, the facilitator rearranges the constellation until a so-called solution picture emerges. It is defined by the AC’s perception of experiencing a social system that has changed for the better. The goal of a SC is to help the client gaining insights into, understand, and finally change his or her inner image of a conflictive systemic dynamic, for example, a dysfunctional relationship with a team member, through the experience of proceeding through several steps from an initial problem constellation to a closing solution constellation [14, 15].

### Virtual reality (VR)

VR is a form of human–computer interaction in which the human being uses a virtual output device which is placed on the head, the head mounted display, to become an AC in a computer-generated 3-dimensional (3D) environment. The experience of participation is intensified by sensors that react to head and body movements. In addition, the use of manual control elements, the hand controllers, opens further possibilities for interactions with the virtual world, the objects in it, and other participants [16]. The aim is to achieve the highest possible level of immersion, that is covering the senses with virtual sensory impressions, and thus achieving the most intensive subjective feeling of immersion [17]. The VR technology is being used successfully in cognitive behavioral therapy, especially for the treatment of phobias and anxiety disorders [6]. The purpose of this study is to investigate the feasibility of SCs in VR as a systemic intervention, e.g., for people with chronic sociopsychological conflicts.

### System constellation in VR

Most formats of SCs have in common, that they require a group setting in presence, usually with more than 10 people. Clients, facilitators, potential representatives and observers must come together in one place, usually in the context of 1- to 3-day constellation seminars [2–5]. This requires a large logistical, time and financial effort, and thus makes it more difficult to use this form of intervention in everyday practice. An easier access could maybe provided via VR. In addition, VR opens up possibilities that are difficult to realize in face-to-face settings, e.g., reducing or enlarging objects, letting objects float, choosing from different virtual locations, or observing the system constellation from new angles like bird’s eye view. This opens up new possibilities for the implementation and further development of SCs when transferred in VR. The previously mentioned phenomenon of *immersion* also suggests that VR could be superior to the online setting in certain aspects [18]. Firstly, it is to be assumed that the participants’ level of *presence* and *engagement* is increased, making them feel more involved and improving the effectiveness of the setup. Secondly, immersive VR could lead to a *deeper emotional experience* as participants could identify more strongly with the presented systems and dynamics, allowing hidden emotions and conflicts to be brought to light and deepening the constellation process. Furthermore, immersion may enable participants to freely move and interact with elements in the virtual environment, facilitating exploration of different perspectives, visualization, and understanding of relationships between system components. Additionally, regarding the immersive nature of VR, it can be assumed that the clients’ attention and focus are increased, as they might be less distracted and could concentrate on the setup and dynamics [19]. In our study, the clients’ experiences with VR will be documented through observations during the system constellation seminars, the use of self-report questionnaires [20–22], and through qualitative interviews after the seminars.

### Efficacy

Evidence-based research on SCs appear marginal. This is despite the fact that SCs are well implemented and often applied in psychiatric, psychological, and psychotherapeutic settings as well as in different contexts of organizational counseling. The “Heidelberg study on systemic constellations” [2], the first RCT in this field, investigated (1) the short-term efficacy of SCs compared to a wait-list group 2 weeks and 4 months after participation in a SC (Study 1, *n* = 208), (2) the medium-term efficacy for the intervention group after 8 and 12 months (Study 2, *n* = 104), and (3) the long-term efficacy after 5 years cumulated for the intervention and wait-list group with subsequent intervention (Study 3, *n* = 137). Clients were mainly midlife women (*M* = 47 years; *SD* = 9; 84% female), married or living with a partner (66%), highly educated (89%), employed (96%), and with previous experience in SCs (80%). Analyses of variance (ANOVAs) with repeated measures and analyses of simple effects within and between groups were performed. In Study 1, after 2 weeks, and with stable effects compared to the wait-list group after 4 months, results showed significant attainment on SC-related goals classified according to the taxonomy of the Bern Inventory of Treatment Goals (BIT-T) [23, 24] and significant improvement on psychological functioning with a small to medium effect size (*d* = 0.45–0.51), including the Outcome Questionnaire (OQ-45.2) [25, 26], and the psychological distress using the Questionnaire for the Evaluation of Treatment Progress (FEP-2) [27]. It also showed significant improvement on interpersonal functioning with a medium effect size (*d* = 0.52–0.55), including the incongruence questionnaire [28]. Significant improvements also emerged on systemic functioning, again with a medium effect size (*d* = 0.53–0.61), including the Experience in Personal Social Systems Questionnaire (EXIS) [29]. In study 2, the psychological functioning and psychological distress stayed stable after 8 and 12 months with small to a medium effect size (*d* = 0.39–0.50), interpersonal functioning with a small effect size (*d* = 0.35–0.44), and systemic functioning with a medium effect size (*d* = 0.57–0.61). In study 3, at 5-year follow-up, the psychological functioning decreased to the level measured at baseline (*d* = 0.10–0.15), whereas the systemic functioning demonstrated a stable effect (*d* = 0.48) [2–5].

### Aims and objectives

As this study is the first trial to investigate SCs in VR, we start with a prospective, monocentric, parallel-group RCT with subsequent intervention. The aim is to explore the feasibility and acceptability of the SC-VR intervention to carefully plan a fully powered RCT, to see whether it can be implemented with success [30], to explore potential effects, and if so, to calculate the number of clients needed to replicate effects indicated in this study [31]. For that purpose, we descriptively and exploratively investigate the following research questions (RQ).

1. Feasibility and acceptability of the research methodology
  RQ1.1: Is it possible to recruit the planned number of clients?
  RQ1.2: How many clients must be recruited, will participate according to the study protocol or show study protocol violations, and what is the retention rate?
  RQ1.3: How well are the outcome variables accepted by the clients?
2. Feasibility and acceptability of the SC-VR intervention
  RQ2.1: How well fits the VR environment the needs of SCs?
  RQ2.2: How comparable is the facilitation of SCs in VR to SCs in presence, and do we have to consider specific differences?
  RQ2.3: What is the degree of the facilitator’s adherence to SCs in VR, i.e., the percentage of treatment components defined by the manual across each facilitation and seminar that were implemented as planned?
  RQ2.4: How strong is the clients’ compliance with VR?
  RQ2.5: How do the clients and facilitators will rate the ease and intensity of participating in both the SC and the VR environment, including potential cybersickness and the level of immersion, and the clients’ expectancy of the SC-VR seminar?
  RQ2.6: What are additional identified benefits and barriers of VR for the use of SCs?
  RQ2.7: Will there be any adverse event reported by the clients throughout the study period?
3. Estimation of effect sizes
  RQ3.1: How likely is a potential effect of SCs in VR to relate to the SC versus the VR environment?
  RQ3.2: What is the estimated within-subjects effect size and 95% CI for changes in the secondary outcome, i.e., psychological functioning, from baseline to 4-month follow-up?
  RQ3.3: What are the within-subjects effect sizes and 95% CI for changes for the secondary outcomes, i.e., psychological distress, systemic functioning, motivational incongruence, and goal attainment, from baseline to 4-month follow-up?
  RQ3.4: What proportion of clients experience which clinically meaningful change, i.e., remission, response, deterioration, and no change?

## Methods

### Design

The study is designed as a prospective, monocentric, parallel-group, feasibility RCT with subsequent intervention. A total of 128 clients of group-based SCs in VR-seminars will be randomized to either the intervention group (IG, *n* = 64) or wait-list group (WLG, *n* = 64). Each study facilitator will conduct four SC-VR seminars in tandem-partnership. The short-term intervention outcome will be measured including three assessment points: baseline and 2-week and 4-month follow-up, with the latter representing the primary endpoint. In addition, we will assess mid-term effects at 8-month follow-up and long-term effects at 12-month follow-up.

### Ethical consideration

SC-VR seminars will be practiced in a decentralized manner. Facilitators and clients will take part via VR headset from self-selected places. The feasibility study design, the measurement times, and instruments are aligned with the “Heidelberg study on systemic constellations” [2–5]. This research is approved by the UW/H Ethics Committee (S-232/2021) and registered with clinical trials (www.clinicaltrials.gov; ClinicalTrials.gov: ID = NCT05557890). Written informed consent will be obtained from each interested person. Staff will send the study information, informed consent, and declaration about the usage of recorded data to each interested person after the initial telephone interview. The staff will be available for any questions on the telephone or via videoconference during workdays. All interested persons will be informed about their rights to end their participation at any time without negative consequences. The Data Monitoring Committee (DMC) is part of the Chair of Clinical Psychology and Psychotherapy III, Witten/Herdecke University. It is independent from any sponsors and competing interests. Confidentiality will be maintained at all levels of the feasibility study by staff members, facilitators, and researchers. All of them must declare bindingly that they will give no information to third persons. Additionally, we will work with identification codes for each interested person and client. All data will be saved pseudonymized on the UW/H server. This feasibility trial will be conducted in accordance with the Declaration of Helsinki [32]. Data management procedures can be found in the approval of the UW/H Ethics Committee (S-232/2021). The results of this feasibility study will be published only on the group mean value in peer-reviewed journals and at academic conferences. To enhance transparency and the quality of reporting, findings will be presented in accordance with the CONSORT 2010 extended statement for randomized pilot and feasibility trials [33].

### Protocol amendments

Modifications to the study protocol may impact the conduct of the study. This encompasses alterations to the study design, investigators, objectives, sample size, procedures, and data collection forms. Any such changes will be formally submitted to the UW/H Ethics Committee following a comprehensive discussion and consensus between the principal investigator, co-investigators, and the research coordinator. Participants will be informed of any changes that may affect their participation in the study. It should be noted that changes that do not affect the conduct of the study, such as minor protocol corrections or administrative changes, will not be communicated to participants.

### Study procedures

**Fig. 1:**
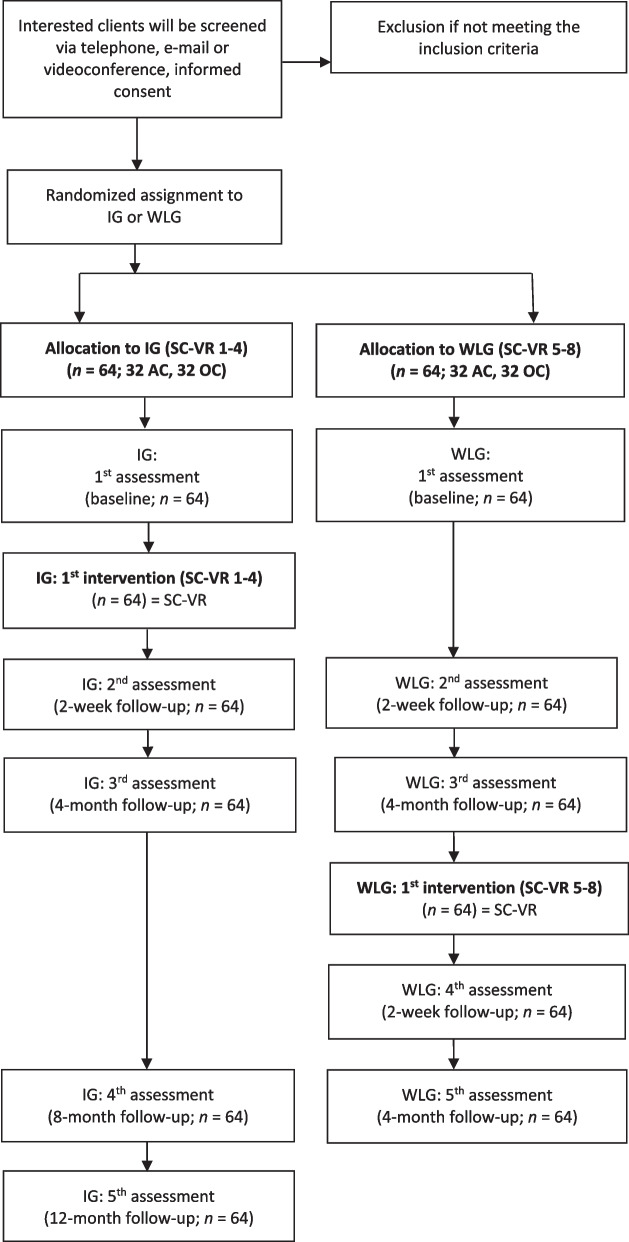
Design, assessments and client flow. *IG* intervention group, *SC-VR* system constellation in virtual reality, *WLG* wait-list group, *AC* active clients, *OC* observing clients

In the screening phase, interested persons will receive the study information and will be asked for the fulfillment of inclusion and exclusion criteria. The final decision whether the clients fulfil the inclusion criteria will be made in case conferences by the research team at the UW/H. After inclusion, clients will be randomly assigned either to the IG or WLG. Subsequently, they will be informed in which group they will attend the SC. About 1 week before the SC-VR seminar, the client will get their VR equipment via post. This includes an onboarding manual for setting up the VR headset and using the hand controller in an VR environment. About 2 days before the SC-VR seminar, there will be an individualized onboarding, partly via videoconference, in which the clients will get access and explore the VR seminar environment. We will assess study outcomes before the start (baseline) of the SC-VR seminar, at 2-week, 4-, 8- and 12-month follow-up. Baseline data will be scheduled no more than 5 days before the first SC-VR seminar (Fig. 1) and (Table 1).

**Table 1:** Assessment measures and application plan

**Purpose**	**Perspective**	**Domain**	**Measure**	**Baseline**	**SC-VR seminar **	**2-week ** **follow** **-up**^**a**^	**4-month ** **follow** **-up**	**8-month ** **follow-up**	**12-month ** **follow-up**^**b**^
Prim. Outc.	Client	SC-VR Adherence	Manual		X				
Sec. Outc.	Client	Psychological well-being	OQ-45.2	X		X	X	X	X
Sec. Outc.	Client	Social system functioning	EXIS	X		X	X	X	X
Sec. Outc.	Client	Intervention Outcome	FEP-2	X		X	X	X	X
Sec. Outc.	Client	Goal Attainment	BIT-T	X		X	X	X	X
Sec. Outc.	Client	Motivational Incongruence	INK-SF	X		X	X	X	X

*Prim. Outc.* Primary outcome, *Sec. Outc.* Secondary outcome, *OQ-45.2* Outcome Questionnaire, *EXIS *Experience in Social Systems, *FEP-2 *Questionnaire for the Evaluation of Treatment Progress, *BIT-T *Bern Inventory of Psychotherapy Goals, *INK-SF *Incongruence Questionnaire, short-form, *SC-VR *System constellation in virtual reality

^a^2-week follow-up will be assessed post SC-VR in the intervention group for both the intervention and wait-list group, and at 2-week follow-up post SC-VR in the wait-list group only for the wait-list group

^b^12-month follow-up will be assessed only for the intervention group

### VR technology

The technological setup for the study consists of the application RAUM in combination with the VR HDM Meta Oculus Quest II. RAUM (https://www.raum.app/) is a commercial VR collaboration platform with full spatial interaction and tools designed to be used in three dimensions. An unlimited screenspace and high-quality graphics enable a fully immersive experience. RAUM comes with a set of different virtual locations. However, for the study, we use a minimalistic virtual room setting called “campus” with lines on the floor framing the field for the SCs. Each participant can design his own avatar or choose from a set of pre-made avatars. The VR HMD Oculus Quest II (https://www.meta.com/quest/products/quest-2/) is a commercially available VR HMD with two hand-operated controllers which only needs a Wi-Fi connection to access the RAUM app. For using RAUM properly, a 1.5 Mbps upload and a 5 Mbps download speed per user are recommended. The HMD detects head movement, and the controllers track hand movements via 3D inertial sensor technology.

### Sample size calculation

Although feasibility studies, e.g., to develop an intervention, do not require a sample size calculation, it was important for us to ground the number of participants in our study on a statistical rationale that would allow a statement to be made about the sample size needed for a confirmatory trial. Cocks and Torgerson [31] used a confidence interval (CI) approach to calculate pilot sample sizes for continuous outcome measures. Supposed 0.3 of a standard deviation between two groups would be worthwhile, then such a study requires about 350 clients (assuming 80% power, two-sided alpha of 5%) in the final analysis of the fully powered RCT. Consequently, 32 clients (approximately 9% of the main sample size) would be required to produce a one-sided 80% confidence limit. A pilot trial with that sample size which finds an estimate larger than zero is supposed to demonstrate feasibility of a confirmatory RCT considering the recruitment and retainment of clients and so forth. According to this approach for two-arm feasibility RCTs, we will recruit a minimum of 32 clients. Since we are in the good position of sufficient personal and financial resources, we can include more clients in our study, and if possible, we will recruit 128 individuals, i.e., 64 clients in the IG and WLG, respectively (Fig. 1).

### Recruitment

#### Clients

Recruitment of clients will start in autumn 2023 and end when we have included 128 clients which is expected in winter 2024. We will distribute study information on the UW/H website, on information portals, on clinical trials, via e-mail lists of the Witten Institute of Family Business (WIFU), and social media. All clients will present themselves to the study team, and no client will be referred.

#### Facilitators

Recruitment of facilitators started in winter 2022 and ended in summer 2023. The information was distributed to the network of TVB and CHS. All facilitators presented themselves to the study team and gave consent to take part in trainings to practice the VR technology for SCs. All facilitators meet the quality standards of the German Society for System Constellations (DGfS) and/or the International Forum for System Constellations in Organizations (infosyon), in addition to several years of counseling experience.

### Inclusion and exclusion criteria

#### Clients

To contribute to the external validity by designing SC seminars in our study similar to those carried out in naturalistic settings, the inclusion criteria will be minimal. Persons of either gender who are at least 18 years or older can take part if they (a) present with a psychosocial conflict that they have not been able to resolve for some time: these can be chronic conflicts in private social systems (e.g., partnership, family, friendships) as well as in organizational social systems (e.g., team, employee-supervisor) or professional social systems (e.g., care networks). They will be included if they (b) will participate in a 3-day SC-VR seminar, (c) will make their own decision to participate as active client (AC) or observing client (OC), (d) will accept random allocation to the IG or WLG, (e) will agree to randomized assignment to the study facilitators, and will abstain from taking part in any other SC until completion of the study. To avoid harm, clients will be excluded from the intervention if they show (a) acute suicidal tendencies, (b) an acute psychotic episode, or (c) an acute drug or alcohol intoxication. No previous experience with SC and/or psychotherapy, as well as VR, is needed either as an inclusion nor exclusion criteria.

#### Facilitators

To ensure the quality of the interventions and the competency to deal with study clients with chronic sociopsychological conflicts, eligibility criteria for the facilitators encompass (a) the certification as facilitator according to the quality standards of the German Society for System Constellations (DGfS) or the International Forum for Organizational Constellations (infosyon), (b) professional experience in counseling or coaching of at least 5 years, and (c) consent to take part at a minimum in a 4- to 5- day training using VR technology and applying SC in VR.

### Randomization

Following inclusion, clients will be randomized using restricted randomization to obtain a balanced sample size between groups for each seminar. Therefore, we will use a randomization plan generator (www.randomization.com). The random allocation rule will be implemented according to the restricted shuffled approach, a kind of block randomization which we will perform in a 1:1 ratio [34]: every 2 people will be statistically paired and randomly assigned to either the IG (*n* = 64) or WLG (*n* = 64), after stratification by role, i.e., voluntary choice to participate either as an AC or OC prior to randomization. The randomization procedure will be performed by an independent researcher who is not involved in the study.

### Blinding

#### Clients

They will not be informed about the design of this RCT, whether they will be assigned to the IG or WLG, and the specific study research questions. They will be informed about their randomized assignment to any of the SC-VR seminars. The cover story includes that the dates of the SC-VR seminars are as they were provided by the facilitators. It is not made public that the earlier group of SC-VR seminars refers to the IG and the later group of SC-VR seminars to the WLG. This procedure is closely related to the way in which participants come to SC seminars in natural settings. After study completion, all clients will be unblinded, and the design of the study will be published. All assessments at all points in time will be conducted online and pseudonymized, and no blinding of assessors will be needed.

#### Facilitators

The facilitators are informed about the study design as the original RCT [2–5] is well-known amongst them. Consequently, blinding was not possible without jeopardizing the facilitators’ trust in the study team.

### Facilitator’s training and supervision

All facilitators will participate in a 4- to 5-day training using VR technology and applying it to SC. The SC in VR technology trainings will take place in autum 2023 and will be conducted by a professional VR training institute: RAUM virtual collaboration and CONENT Conscious Entrepreneurship.

### Measures

As the purpose of this study is to explore the feasibility of a fully powered RCT, we will use different measures, including both qualitative information and quantitative data.

#### Feasibility and acceptability measures

##### Research methodology

- RQ1.1 and RQ1.2: The feasibility of protocol implementation will include the ability to meet the total recruitment goal (*n* = 128), in the IG (*n* = 64) and WLG (*n* = 64) in particular. It takes the estimation of the total sample to be recruited into account, the total drop-out rate, and differential drop-out rate within the study arms at all measurement times. Data will be collected through the observation of the client flow: counting the number of interested persons who will (1) contact the recruiting office, (2) drop out by not giving consent, (3) drop out before the beginning of the SC-VR seminars, (4) start SC-VR seminar, (5) drop out during the SC-VR seminars, (6) end SC-VR seminar, (7) participate in the 2-week follow-up, (8) participate in the 4-month follow-up, (9) participate in the 8-month follow-up, and (10) participate in the 12-month follow-up. All clients must be abstained from taking part in any another SC until completion of the study. Success will be defined as the achievement of 100% of the planned sample and retaining 85% of the clients in the SC-VR intervention.
- RQ1.3: Outcome measure appropriateness will be assessed through the observation of missing data and the delay in answering the outcome measures. Success will be defined by missing data below 20% of the total data set and the answering of outcome measures within 3 days around the measurement date.

##### SC-VR intervention

- RQ2.1 and RQ2.2: The feasibility and acceptability of the SC-VR intervention will be questioned in semi-structured experience-based interviews [35] considering the appropriateness of the VR environment to the needs of SCs, differences in comparison to SCs in presence, and whether there have to be considered specific adaptations to the VR environment.
- RQ2.3: Facilitator adherence will be evaluated by two independent raters for each SC-VR seminar using the SC adherence scale from the “Heidelberg study on systemic constellations” [2–5].
- RQ2.4: Clients’ compliance will be assessed by the percentage of seminars attempted and completed.
- RQ2.5: The ease and intensity of participating in both the SC and in the VR environment, and the enjoyment of the SC-VR seminar, will be assessed based on the potential cybersickness using a validated 7-item scale [36], and the level of immersion [37] in the VR experience using a validated 15-item measure. In addition, we will measure clients’ expectancy using a validated 5-item scale [38].
- RQ2.6: Additional identified benefits and barriers of VR for the use of SCs will be questioned in semi-structured experience-based interviews [35].
- RQ2.7: Finally, as part of good clinical practice, adverse events will be monitored throughout the study and considered in relation to intervention safety and potential adverse outcomes. For any adverse event during or after the intervention, psychological psychotherapists at the Center for Mental Health and Psychotherapy (ZPP) of UW/H, co-directed by Prof. Dr. Christina Hunger-Schoppe, will be available.

##### Estimation of effect sizes

- RQ3.1: The potential effect of SCs in VR to relate to the SCs versus the VR environment will be questioned in semi-structured experience-based interviews [35].
- RQ3.2: The estimation of effect sizes and 95% CI for change will concentrate on the proposed secondary outcome, i.e., psychological functioning (OQ-45.2), from baseline to 4-month follow-up.
- RQ3.3: In addition, effect sizes and 95% CI for change in the proposed secondary outcomes, i.e., systemic functioning (EXIS), psychological distress (FEP-2), motivational incongruence (INK-SF), and goal attainment (GAS), will be calculated from baseline to 4-month follow-up.
- RQ3.4: The proportion of clients experiencing clinically meaningful change, i.e., remission, response, deterioration, and no change, will be explored for the proposed secondary outcome (OQ-45.2)

#### Proposed primary outcome measure

For research methodology feasibility measures, we will calculate screening, recruitment, randomization, and drop-out rates (RQ1.1, RQ1.2) as well as missing data in answering the outcome measures (RQ1.3). For intervention feasibility measures, we will use qualitative content analysis (QCA) [39] and/or Consensual Qualitative Research (CQR) [40, 41] to inform about the appropriateness of the VR environment to the needs of SCs, and benefits as well as barriers of VR for the use of SCs (RQ2.1, RQ2.2, RQ2.6). The facilitator’s manual adherence will be calculated using Cohen’s kappa as a robust measure of interrater reliability, in addition to the percentage (%) of the agreement between the two raters. Overall treatment integrity for each SC-VR will be calculated as the percentage (%) of treatment components defined by the manual across sessions that will be implemented as planned [42] (RQ2.3). Clients’ compliance will be assessed by the percentage (%) of seminars attempted and completed (RQ2.4). The clients’ expectancy of the SC-VR seminar, as well as potential cybersickness and the level of immersion in the VR experience, will be assessed by mean scores (M), standard deviations (SD), and 95% CI of the data gathered by using a validated 5-item [38], 7-item [36], and 15-item [37] measure (RQ2.5). Adverse events will be monitored and reported descriptively (RQ2.7).

#### Proposed secondary outcome measures

The *Outcome Questionnaire* (OQ-45.2) [25, 26] will be used to assess psychological functioning. The OQ-45.2 is a self reporting instrument that measures psychotherapeutic change over the previous week. Its total score (OQ-TOT) indicates symptom distress, quality of interpersonal relations, and social role performance. The OQ-45.2 features test–retest reliability of 0.84, excellent internal consistency at 0.93, sensitivity to change, and concurrent validity with a variety of self-report scales [25, 43, 44].

The *Questionnaire for the Evaluation of Treatment Progress (FEP-2)* [27] will be used for measuring psychological distress. The FEP-2 measures how persons feel over the previous week. Its total score (FEP-TOT) indicates well-being, symptom distress, interpersonal relationships, and congruence. The FEP-2 shows test–retest reliability of 0.77 (1 week) and internal consistency of 0.94. Concurrent validity and sensitivity to change were demonstrated.

The *Incongruence Questionnaire (INK-SF)* [28] will be used to measure motivational incongruence. Its total score (INK-SF-TOT) indicates approach goals (the maximization of desirable outcomes) and avoidance goals (the minimization of unwanted outcomes). One-week test–retest reliability was 0.81, and internal consistencies ranged from 0.75 to 0.91. Criterion validity was assessed using established instruments for psychopathological symptoms and quality of life.

The *Experience in Social Systems Questionnaire (EXIS)* [29] measures basic dimensions of what SCs aim to change in how clients experience themselves within their most important social and organizational systems (I-within-my-systems). The total score measures the clients’ experience in their personal systems (EXIS.pers) or organizational social systems (EXIS.org) containing the subdimensions of experiencing belonging, autonomy, accord, and confidence. The EXIS shows an internal consistency of 0.90. Concurrent validity and sensitivity to change were demonstrated.

A 4-point Likert scale (1 = not attained; 2 = partially attained, 3 = attained, 4 = fully attained) will be used to assess *goal attainment (GA*). At baseline, all study clients will be asked to identify their goal for the SC-VR in free text. Subsequently, clients’ goals will be classified according to the taxonomy of the Bern Inventory of Treatment Goals (BIT-T) [23] by the research team. The BIT-T encompasses five categories: coping with specific problems and symptoms (P), interpersonal goals (I), well-being and functioning (W), existential issues (E), and personal growth (G). In (re-)coding an extended sample of client treatment goals, the BIT-T proved to have a good interrater reliability, identified differences between diagnostic groups, and showed meaningful relations to standardized intake measures [24].

Clients will complete a brief *demographic measure*, including age, sex, education level, and whether they are part of a business family or a family business.

### Adverse events

Harm will be assessed by means of passive surveillance [45]. Clients will be instructed to contact the facilitators or any study team member whenever they will experience adverse events, defined as any unfavorable change in physical and/or mental condition at any time between allocation, end of the intervention, and end of study period. If necessary, clients will receive psychosocial support, including psychotherapy, in the cooperating psychotherapy outpatient Center for Mental Health and Psychotherapy (ZPP) of UW/H.

### Analysis

There will be no interim analyses. The only stopping guideline refers to the stop of recruitment 4 weeks before the start of the intervention due to organizational needs. Even if the number of participants is not reached at this time point, there will be no further recruitment at a later date. For *research methodology feasibility* measures, we will calculate screening, recruitment, randomization, and drop-out rates (RQ1.1, RQ1.2) as well as missing data in answering the outcome measures (RQ1.3) at baseline, 2-week, and 4-, 8-, and 12-month follow-up.

For *intervention feasibility* measures, we will use QCA and/or CQR to inform about the appropriateness of the VR environment to the needs of SCs and benefits as well as barriers of VR for the use of SCs (RQ2.1, RQ2.2, RQ2.6). The facilitator’s manual adherence will be calculated using *Cohen’s kappa* as a robust measure of interrater reliability, in addition to the percentage *(%)* of the agreement between the two raters. Overall treatment integrity for each SC-VR will be calculated as the percentage *(%)* of treatment components defined by the manual across sessions that will be implemented as planned [42] (RQ2.3). Clients’ compliance will be assessed by the percentage *(%)* of seminars attempted and completed (RQ2.4). The clients’ expectancy of the SC-VR seminar, as well as potential cybersickness and the level of immersion in the VR experience, will be assessed by mean scores (*M*), standard deviations (*SD*), and 95% *CI* of the data gathered (RQ2.5). Adverse events will be monitored and reported descriptively (RQ2.7).

For *estimation of effect sizes*, we will use QCA and/or CQR to analyze information about the association of these effects to either the SCs or the VR environment, or both (RQ3.1). Because clients within groups (i.e., SC-VR seminar) may be more similar to each other than clients in different SC-VR seminars, a two-level linear regression analysis will be performed to account for potential clustering effects at higher levels (i.e., clients nested within SC-VR seminars). The intraclass correlation (ICC) coefficient from the random intercept model with clients (level 1) and SC-VR seminar (level 2) will be calculated for the proposed secondary outcome (OQ-45.2) [46]. An ICC greater than zero will indicate a clustering effect, and any statistical analysis must be adjusted for this effect. All proposed outcomes will be analyzed as intention-to-treat using mean scores (M), standard deviations (SD), and 95% CI. Missing values of less than 20% will be replaced with the conditional mean value of the four subgroups (ACs in IG, and WLG; OCs in IG, and WLG). This will be compared to per-protocol analyses. We then will use mixed-design ANOVAs (group: IG, WLG; client status: AC, OC; time: baseline, 2-week and 4-month follow-up), adjusting for baseline scores considering demography as well as outcome data. Because treatment outcome will be measured at three assessment points, within-group effects will be further analyzed by comparisons between baseline and 2-week follow-up (contrast A), and by comparisons between 2-week and 4-month follow-ups (contrast B). Effect sizes will be presented as partial eta-squared values (η^2^) and Cohen’s *d*, calculated as the difference between the means divided by the pooled standard deviation $(d=\frac{{x}_{1}^{2}-{x}_{1}^{2}}{\frac{\sqrt{{s}_{1}^{2}+{s}_{2}^{2}}}{2}})$. Classification of *effect sizes* will be as follows: η^2^ ≥ 0.010, small effect; η^2^ ≥ 0.060, medium effect; η^2^ ≥ 0.140, large effect; Cohen’s *d* ≥ 0.20, small effect; *d* ≥ 0.50, medium effect; and *d* ≥ 0.80, large effect [47] (RQ3.2, RQ3.3). The exploration of *reliably significant change*, i.e., the proportion of remission, response, deterioration, and no change, will be limited to the proposed secondary outcome (OQ-45.2) based on the reliable change index (RCI) [48]. We will use previously established internal consistency reliabilities to calculate the RCI [49] and Jacobson and Truax’s [48] cutoff C for the categorization of the clients’ (no) change [26–28] (RQ3.4).

For *qualitative data analyses*, we collect qualitative information for in-depth analysis of the appropriateness of the VR environment to the needs of SCs, benefits as well as barriers of VR for the use of SCs (RQ2.1, RQ2.2, RQ2.6), and to analyze information about the association of SC-VR effects to either the SCs or the VR environment (RQ3.1). We will perform semi-structured interviews with the participants. The qualitative data will be interpreted based on QCA [37] and/or CQR [43].

## Discussion

To the best of our knowledge, this will be the first study to explore the feasibility of SCs in VR in a prospective, monocentric, parallel-group, feasibility RCT with subsequent intervention. The challenge is to apply SC in VR as this technology has not yet reached the mainstream, so facilitators and clients must be familiarized with this innovative environment.

### Innovative aspects

What most formats of SCs have in common is that they require a group setting in the presence with 10 or more clients. Therefore, the intervention usually takes place within 1- to 3-day SC seminars. This requires greater logistical, time, and economic investments, which makes it difficult to use SCs regularly in everyday counseling. The in-person setting also increases the barrier for people who want to work on their personal issue, i.e., chronic psychosocial conflicts, as anonymously as possible. A new, expanded and possibly lower-threshold approach might be the participation in a SC in VR.

### Biases and limitations

This trial is a feasibility study, and all statistical analyses will be descriptive and explorative, with the aim to obtain data that can then be used for planning a subsequent confirmatory RCT. Consequently, the main RCT is necessary before any confirmatory statement about the efficacy of SCs in VR can reliably be made. We will use a WLG design instead of an active control group treatment [50]. We will select only four study facilitators: their long-term experience and expertise in conducting SCs also limits generalizability. Their allegiance to the treatment, a factor known to contribute to the outcome regardless of the specific intervention [51], may be high. Therefore, our results have to be discussed in terms of facilitator allegiance. Although investigators will not proponents of the SC approach, four of the six authors are systemic therapists or counselors; thus, their presence as researchers in the SCs can also influence the group process and the proposed outcomes.

### Perspectives of a confirmatory trial

We strive for a subsequent confirmatory multicentric RCT comparing SC-VR seminars either in an RCT design with subsequent intervention or using an active control group. We believe that the proposed outcome measures we will use in this feasibility RCT can also be used in other RCTs, as they have already been proven in previous investigations on SCs [2–5]. In addition to the very experienced SC facilitators in our study, we hope to integrate facilitators from various levels in future studies. It however is important to point out that while the facilitators in our study are experienced in SC, they are not experienced in VR. Therefore, it would be equally worthwhile to include the same facilitators in the subsequent RCT as they will significantly expand their experience in conducting SCs in VR in this feasibility RCT.

## Data Availability

All relevant data can be obtained by the first and last author (TVB, CHS).

## Abbreviations

ANOVA: Analysis of variance
AC: Active client
BIT-T: Bern Inventory of Treatment Goals
CI: Confidence interval
CHS: Christina Hunger-Schoppe
CQR: Consensual Qualitative Research
DGfS: German Society for System Constellations
EHT: Eik-Henning Tappe
EXIS: Experience in Social Systems
HK: Heiko Kleve
FEP-2: Questionnaire for the Evaluation of Treatment Progress
ICC: Intraclass correlation
IG: Intervention group
INFOSYON: International Forum for System Constellations in Organizations
INK-SF: Incongruence questionnaire (short form)
OC: Observing client
OQ-45.2: Outcome Questionnaire
QCA: Qualitative Content Analysis
RCI: Reliable change index
RCT: Randomized controlled trial
SC: System constellation
SC-VR: System constellation in virtual reality
TD: Thomas Druyen
TR: Tom Rüsen
TVB: Tobias van Bebber
UW/H: Witten/Herdecke University
VR: Virtual reality
WIFU: Witten Institute for Family Business
WLG: Wait-list group
ZPP: Center for Mental Health and Psychotherapy

## References

[1] Corey G. Theory and Practice of Group Counseling: Brooks/Cole Cengage Learning; 2012.

[2] Weinhold J, Hunger C, Bornhäuser A, Link L, Rochon J, Wild B, et al. Family constellation seminars improve psychological functioning in a general population sample: results of a randomized controlled trial. J Couns Psychol. 2013;60(4):601–9. 23957767 10.1037/a0033539

[3] Hunger C, Bornhäuser A, Link L, Schweitzer J, Weinhold J. Improving experience in personal social systems through family constellation seminars: results of a randomized controlled trial. Fam Process. 2014;53(2):288–306. 24251855 10.1111/famp.12051

[4] Hunger C, Weinhold J, Bornhäuser A, Link L, Schweitzer J. Mid- and long-term effects of family constellation seminars in a general population sample: 8- and 12-month follow-up. Fam Process. 2015;54(2):344–58. 25264190 10.1111/famp.12102

[5] Hunger-Schoppe C. Familienaufstellung als Einzelintervention im Gruppensetting bei chronisch-psychosozialen Konflikten: Kurz-, mittel- und langfristige Wirksamkeit. Z Psychiatr Psychol Psychother. 2020;68(4):263–73.

[6] Moessner M, Bauer S. E-Mental-Health und internetbasierte Psychotherapie. Psychotherapeut. 2017;62(3):251–66.

[7] Müller-Christ G. Systemaufstellungen als Instrument der qualitativen Sozialforschung. Vier vielleicht neue Unterscheidungen aus der Anwendungssicht der Wissenschaft. Organisationsaufstellungen: Grundlagen, Settings, Anwendungsfelder. 2016;1:72–93.

[8] Müller-Christ G, Pijetlovic D. Komplexe Systeme lesen. Aufstellungen in Wissenschaft und. Berlin Heidelberg: Springer; 2018.

[9] SCHNEIDER J. Family constellations. Carl-Auer, Heidelberg; 2009.

[10] Cohen DB. “Family constellations”: an innovative systemic phenomenological group process from Germany. Fam J. 2006;14:226–33.

[11] Boszormenyi-Nagy I, Spark GM. Invisible loyalties : reciprocity in intergenerational family therapy. New York (N.Y.): Harper and Row; 1973.

[12] Moreno JL. Psychodrama, first volume. Beacon House, Beacon New York; 1946.

[13] Duhl FJ, Kantor D, Duhl BS. Learning space, and action in family therapy: a primer of sculpture. Semin Psychiatry. 1973;5(2):167–83. 4803385

[14] Weber G. Zweierlei Glück: die systemische Psychotherapie Bert Hellingers: Carl-Auer, Heidelberg; 1997.

[15] Weber G, Schmidt G, Simon FB. Aufstellungsarbeit revisited:... nach Hellinger?: Carl-Auer, Heidelberg; 2005.

[16] Dörner R, Broll W, Jung B, Grimm P, Göbel M. Einführung in Virtual und Augmented Reality. In: Dörner R, Broll W, Grimm P, Jung B, editors. Virtual und Augmented Reality (VR/AR): Grundlagen und Methoden der Virtuellen und Augmentierten Realität. Springer, Berlin Heidelberg: Berlin; 2019. p. 1–42.

[17] Mühlberger A, Pauli P. Virtuelle realität in der psychotherapie. PiD-Psychotherapie im Dialog. 2011;12(02):143–7.

[18] Breves P, Stein J-P. Cognitive load in immersive media settings: the role of spatial presence and cybersickness. Virtual Reality. 2023;27(2):1077–89.

[19] Hofer M. Presence und Involvement. Nomos, Baden-Baden; 2016.

[20] Makransky G, Lilleholt L, Aaby A. Development and validation of the Multimodal Presence Scale for virtual reality environments: a confirmatory factor analysis and item response theory approach. Comput Hum Behav. 2017;72:276–85.

[21] Kourtesis P, Linnell J, Amir R, Argelaguet F, MacPherson S. Cybersickness in Virtual Reality Questionnaire (CSQ-VR): a validation and comparison against SSQ and VRSQ. Virtual Worlds. 2023;2(1):16–35.

[22] Volkmann T, Wessel D, Jochems N, Franke T. German Translation of the Multimodal Presence Scale; 2018;475–9.

[23] Grosse HM. Was möchten Patienten in ihrer Therapie erreichen? - Die Erfassung von Therapiezielen mit dem Berner Inventar für Therapieziele (BIT). Verhaltenstherapie und psychosoziale Praxis. 2001;33:241–58.

[24] Grosse Holtforth M, Grawe K. Der Inkongruenzfragebogen (INK)—Ein Meßinstrument zur Analyse motivationaler Inkongruenz. Z Klin Psychol Psychother. 2003;32:315–23.

[25] Lambert MJ, Gregersen AT, Burlingame GM. The Outcome Questionnaire-45. The use of psychological testing for treatment planning and outcomes assessment: instruments for adults, Volume 3. 3rd ed. Mahwah: Lawrence Erlbaum Associates Publishers; 2004. p. 191–234.

[26] Lambert MJ, Hannöver W, Nisslmüller K, Richard M, Kordy H. Questionnaire on the results of psychotherapy: reliability and validity of the German translation of the Outcome Questionnaire 45.2 (OQ-45.2). Zeitschrift für Klinische Psychologie und Psychotherapie: Forschung und Praxis. 2002;31:40–6.

[27] Lutz W, Schürch E, Stulz N, Böhnke JR, Schöttke H, Rogner J, et al. Entwicklung und psychometrische Kennwerte des Fragebogens zur Evaluation von Psychotherapieverläufen (FEP). Diagnostica. 2009;55(2):106–16.

[28] Grosse Holtforth M, Grawe K, Özgür T. INK-Inkongruenzfragebogen [INK-Incongruence Questionnaire]. Manual. Bern: Verlag Hans Huber; 2004.

[29] Hunger C, Bornhäuser A, Link L, Geigges J, Voss A, Weinhold J, et al. The Experience in Personal Social Systems Questionnaire (EXIS. pers): development and psychometric properties. Family Process. 2017;56(1):154–70. 26858173 10.1111/famp.12205

[30] Arain M, Campbell MJ, Cooper CL, Lancaster GA. What is a pilot or feasibility study? A review of current practice and editorial policy. BMC Med Res Methodol. 2010;10(1):67. 20637084 10.1186/1471-2288-10-67 PMC2912920

[31] Cocks K, Torgerson DJ. Sample size calculations for pilot randomized trials: a confidence interval approach. J Clin Epidemiol. 2013;66(2):197–201. 23195919 10.1016/j.jclinepi.2012.09.002

[32] Association WM. World Medical Association Declaration of Helsinki: ethical principles for medical research involving human subjects. JAMA. 2013;310(20):2191–4. 24141714 10.1001/jama.2013.281053

[33] Eldridge SM, Chan CL, Campbell MJ, Bond CM, Hopewell S, Thabane L, et al. CONSORT 2010 statement: extension to randomised pilot and feasibility trials. BMJ. 2016;355:i5239. 27777223 10.1136/bmj.i5239 PMC5076380

[34] Schulz KF, Grimes DA. Blinding in randomised trials: hiding who got what. Lancet. 2002;359(9307):696–700. 11879884 10.1016/S0140-6736(02)07816-9

[35] Lamnek S, Krell C. Qualitative Sozialforschung. Weinheim: Beltz; 2016.

[36] Davis S, Nesbitt KV, Nalivaiko E. A systematic review of cybersickness. Proceedings of the 2014 Conference on Interactive Entertainment. 2014;1–9.

[37] Cole J, Crowle S, Austwick G, Slater DH. Exploratory findings with virtual reality for phantom limb pain; from stump motion to agency and analgesia. Disabil Rehabil. 2009;31(10):846–54. 10.1080/09638280802355197 19191061

[38] Borkovec TD, Nau SD. Credibility of analogue therapy rationales. J Behav Therap Exp Psychiatry. 1972;3:257–60.

[39] Mayring P. Evidenztriangulation in der Gesundheitsforschung: Kombination von experimentellen, deskriptiven und inhaltsanalytischen Ansätzen [Evidence triangulation in health research: the combination of experimental, descriptive and content-analytical approaches]. Kölner Zeitschrift für Soziologie und Sozialpsychologie. 2017;69(Suppl 2):415–34.

[40] Hill CE, Knox S. Variations on consensual qualitative research. Essentials of consensual qualitative research. Essentials of qualitative methods. Washington: American Psychological Association; 2021. p. 69–80.

[41] Hill CE, Knox S. Conceptual foundations of consensual qualitative research. Essentials of consensual qualitative research. Essentials of qualitative methods. Washington: American Psychological Association; 2021. p. 3–10.

[42] Perepletchikova F. Treatment integrity: A foundation for evidence-based practice in applied psychology. In: Hagermoser Sanetti LM, Kratochwill TR, Hagermoser Sanetti LM, Kratochwill TR, editors. School psychology book series. Washington: American Psychological Association; 2014. p. 131–57.

[43] Haug S, Puschner B, Lambert MJ, Kordy H. Veränderungsmessung in der Psychotherapie mit dem Ergebnisfragebogen (EB-45) [Assessment of change in psychotherapy with the German version of the Outcome Questionnaire (OQ-45.2).]. Zeitschrift für Differentielle und Diagnostische Psychologie. 2004;25:141–51.

[44] Vermeersch DA, Whipple JL, Lambert MJ, Hawkins EJ, Burchfield CM, Okiishi JC. Outcome Questionnaire: is it sensitive to changes in counseling center clients? J Couns Psychol. 2004;51(1):38.

[45] Papanikolaou PN, Christidi GD, Ioannidis JP. Comparison of evidence on harms of medical interventions in randomized and nonrandomized studies. CMAJ. 2006;174(5):635–41. 16505459 10.1503/cmaj.050873 PMC1389826

[46] Kreft I, de Leeuw J. Introducing multivlevel modeling. Thousand Oaks: Sage Publications, Inc; 1998. p. 149.

[47] Cohen J. A power primer. Psychol Bull. 1992;112:155–9. 19565683 10.1037//0033-2909.112.1.155

[48] Jacobson NS, Truax P. Clinical significance: a statistical approach to defining meaningful change in psychotherapy research. J Consult Clin Psychol. 1991;59(1):12–9. 2002127 10.1037//0022-006x.59.1.12

[49] Bauer S, Lambert MJ, Nielsen SL. Clinical significance methods: a comparison of statistical techniques. J Pers Assess. 2004;82:60–70. 14979835 10.1207/s15327752jpa8201_11

[50] Rifkin A. Randomized controlled trials and psychotherapy research. Am J Psychiatry. 2007;164:7–8. 17202535 10.1176/ajp.2007.164.1.7

[51] Leykin Y, DeRubeis RJ. Allegiance in psychotherapy outcome research: separating association from bias. Clin Psychol Sci Pract. 2009;16:54–65.

